# Prevalence of Low Birth Weight before and after Policy Change to IPTp-SP in Two Selected Hospitals in Southern Nigeria: Eleven-Year Retrospective Analyses

**DOI:** 10.1155/2018/4658106

**Published:** 2018-01-04

**Authors:** Nneka U. Igboeli, Maxwell O. Adibe, Chinwe V. Ukwe, Nze C. Aguwa

**Affiliations:** Department of Clinical Pharmacy and Pharmacy Management, Faculty of Pharmaceutical Sciences, University of Nigeria, Nsukka 410001, Nigeria

## Abstract

**Background:**

In 2005, Nigeria changed its policy on prevention of malaria in pregnancy to intermittent preventive treatment with sulphadoxine-pyrimethamine (IPTp-SP). Indicators of impact of effective prevention and control of malaria on pregnancy (MIP) are low birth weight (LBW) and maternal anaemia by parity. This study determined the prevalence of LBW for different gravidity groups during periods of pre- and postpolicy change to IPTp-SP.

**Methods:**

Eleven-year data were abstracted from the delivery registers of two hospitals. Study outcomes calculated for both pre- (2000–2004) and post-IPTp-SP-policy (2005–2010) years were prevalence of LBW for different gravidity groups and risk of LBW in primigravidae compared to multigravidae.

**Results:**

Out of the 11,496 singleton deliveries recorded within the 11-year period, the prevalence of LBW was significantly higher in primigravidae than in multigravidae for both prepolicy (6.3% versus 4%) and postpolicy (8.6% versus 5.1%) years. The risk of LBW in primigravidae compared to multigravidae increased from 1.62 (1.17–2.23) in the prepolicy years to 1.74 (1.436–2.13) during the postpolicy years.

**Conclusion:**

The study demonstrated that both the prevalence and risk of LBW remained significantly higher in primigravidae even after the change in policy to IPTp-SP.

## 1. Introduction

Malaria infection during pregnancy is a major public health problem in tropical and subtropical regions of the world. In most endemic areas of the world, pregnancy remains a high-risk factor for malaria infection [1]. Malaria in pregnancy is associated with adverse pregnancy outcomes for both the mother and the fetus. These include anaemia, parasitaemia, maternal mortality, LBW, prematurity, intrauterine growth retardation (IUGR), abortion, and stillbirth. In areas of high malaria transmission, the risk of low birth weight approximately doubles if women have placental malaria [2], with the greatest effect in primigravidae. LBW can be due to prematurity or IUGR. The relative contribution of IUGR or preterm delivery in causing low birth weight varies by the level of malaria endemicity [3]. The IUGR effect on LBW could be due to histopathological changes of the infected placenta that interferes with nutrient transport. It can also be due to high-density placental parasitaemia with its associated cellular immune responses that lead to the consumption of glucose and oxygen that would have gone to the fetus [4]. Malaria-associated maternal anaemia may also contribute independently to IUGR [4], most likely through a reduction in oxygen transport to the fetus. Prevention of malaria in pregnancy previously consisted of a weekly or bimonthly chemoprophylaxis with chloroquine (CQ) in West African countries and sulphadoxine-pyrimethamine (SP) in East African countries [5]. A large number of trials have demonstrated the efficacy of such a chemoprophylaxis in preventing LBW, maternal anaemia, and placental malaria infection [6, 7]. Unfortunately, because of the growing resistance of parasites to these drugs and poor compliance of pregnant women with the treatment, there is now reduced efficacy with these earlier strategies. In 1998, it was proposed by WHO that chemoprophylaxis should be replaced with intermittent prophylaxis therapy (IPT) for all pregnant women living in areas of stable malaria transmission [8].

The African Roll Back Malaria (RBM) summit in April 2000 adopted the Abuja declaration. The declaration entailed that regional leaders be committed to ensuring that at least 60% of pregnant women in malaria-endemic communities have access to effective prevention and treatment of malaria by the year 2005 [9]. The summit initiated a three-pronged approach to reducing malaria among pregnant women in sub-Saharan Africa. These approaches include effective case management of malaria infections, use of insecticide-treated nets (ITN), and intermittent preventive treatment in pregnancy (IPTp) in areas of stable transmission. Intermittent preventive treatment in pregnancy (IPTp) consists of the administration of a curative dose of an efficacious antimalarial drug at least twice during the second and third trimesters of pregnancy. This drug administration should be during routinely scheduled antenatal clinic visits, regardless of whether the woman is infected or not, and the drug is administered under supervision during antenatal care [10, 11].

Sulfadoxine-pyrimethamine SP is the drug currently recommended by the WHO and also by the Nigerian Malaria Prevention and Control Guideline for the IPTp strategy because of its safety, efficacy, and other advantages [8, 12, 13]. This intervention is based on two tested malaria control strategies: clearing existing parasites (treatment effect seen in mass drug administration) and preventing new infections (prophylaxis). The Nigerian Federal Ministry of Health, through the National Guidelines and Strategies for malaria prevention and control during pregnancy, adopted IPTp with SP for malaria prevention in pregnancy in 2005 [12]. It was recommended that this intervention should be monitored and evaluated regularly in order to assess its impact on pregnancy outcomes. LBW and maternal anaemia have been chosen as good indicators of the impact of effective malaria prevention and control. This study therefore aimed at evaluating the prevalence of LBW for different gravidity groups during periods of pre- and postpolicy change to IPTp-SP.

## 2. Methods

### 2.1. Study Design

The study was a retrospective study during a period of 11 years (2000–2010).

### 2.2. Setting

Two hospitals in two different geopolitical zones of Nigeria—Bishop Shanahan Hospital (BSH), Nsukka, and General Hospital, Calabar—were conveniently used for the study. BSH is a Catholic Mission Hospital located in the southeast region of Nigeria. It is a 150-bedded hospital that serves the immediate environs. It also has been serving as a referral centre for the surrounding communities since 1930. General Hospital, Calabar, is a government-owned secondary healthcare facility located in the south-south region. It is a 100-bedded hospital that has been in existence since 1991.

### 2.3. Data Collection

All available and legible entries in the delivery registers for the years 2000–2010 were abstracted (2000–2009 for Bishop Shanahan Hospital and 2001–2010 for General Hospital, Calabar). The prevalence of LBW for different gravidity groups during periods of pre- and postpolicy change to IPTp-SP was determined through analysis of retrospective hospital data from delivery registers (birth weight, stillbirths, parity, age, gestation age, twin birth, and delivery outcome). The delivery register data covered a period of five years before SP-IPTp policy (2000–2004) and six years after SP-IPTp policy (2005–2010).

Data were entered directly into an Excel spreadsheet specially prepared to collect relevant data from the registers.

### 2.4. Data Analysis

Birth weights were categorized as LBW (≤2.5 kg) or normal weight (>2.5 kg). Chi-square analysis was used to compare the prevalence of LBW in primigravidae against multigravidae. Logistic regression was used to determine the association between LBW and gravidity during the pre- and post-policy periods. This was done by calculating the odds ratio before and after the policy change and also the yearly odds ratio for the eleven-year period. These analyses were done using SPSS version 16 statistical software.

Furthermore, the significance of the changes between the pre- and postpolicy periods was evaluated for the prevalence of LBW for different gravidity groups using chi-square and for the calculated odds ratio using independent student's *t*-test, respectively. These last analyses were carried out using GraphPad® software. The criterion for statistical significance was set at *p* < 0.05.

## 3. Results

The delivery registers show a total of 11496 mother-baby(ies) delivery pairs between 2000 and 2010 in the two hospitals assessed. Of these, 11249 (97.85%) were singleton deliveries and were used in further analysis of the birth weight data. The mean maternal age in years was 27.90 ± 5.345, while the mean birth weight in kg was 3.26 ± 0.563. The prevalence of LBW among this delivery cohort was 6% (640). Other demographic and obstetric characteristics of mother-baby pairs from the delivery registers are summarized in Table 1.

**Figure 1:**
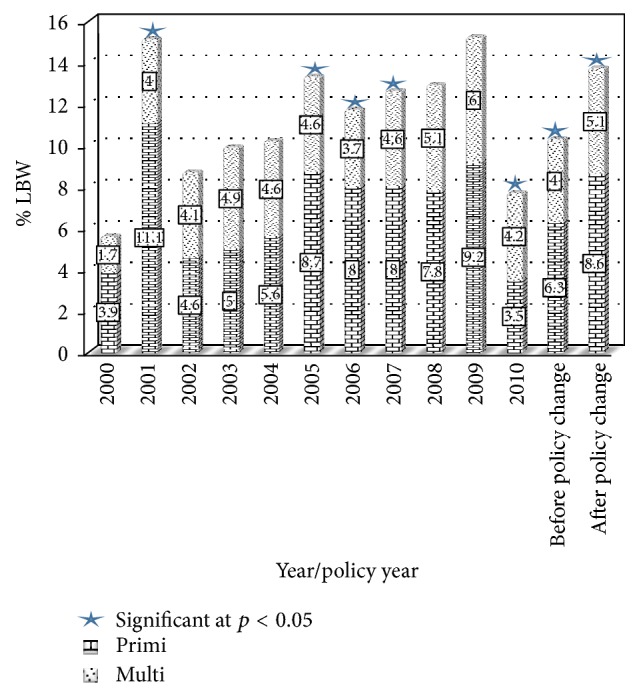
Percentage distribution of low birth weight by year/period of study. Blue star indicates significant difference in the prevalence of low birth weight between primigravidae and multigravidae.

The comparison of prevalence of LBW deliveries between gravidae varied across the years, but higher prevalence was almost consistently noted among primigravidae across the years. On individual year analysis, the prevalence of LBW deliveries during the pre-policy years (2000–2004) was only significantly higher amongst the primigravidae in 2001 (11% versus 4%; *p* < 0.001). On the other hand, the prevalence of LBW deliveries was significantly higher amongst primigravidae in all years except two years, during the postpolicy years (2005–2010) (Figure 1). Presenting the years in year blocks, the primigravidae had significantly higher prevalence of LBW deliveries both before policy change (2000–2004) (6.3% versus 4%; *p* < 0.001) and after policy change (2005–2010) (8.6% versus 5.1%; *p* < 0.001) (Figure 1).

Presenting the years into two blocks, pre-policy years (2000–2004) and post-policy years (2005–2010), Table 2 summarizes the overall odds ratio changes in the pre- and post-policy years. There were significantly increased odds of having LBW deliveries among primigravidae in both pre-policy (OR = 1.62, 95% CI = 1.17–2.23) and post-policy years (OR = 1.74, 95% CI = 1.43–2.13). A non-significant increase in OR [1.62 to 1.74, *p* = 0.7075] was recorded across policy year.

**Figure 2:**
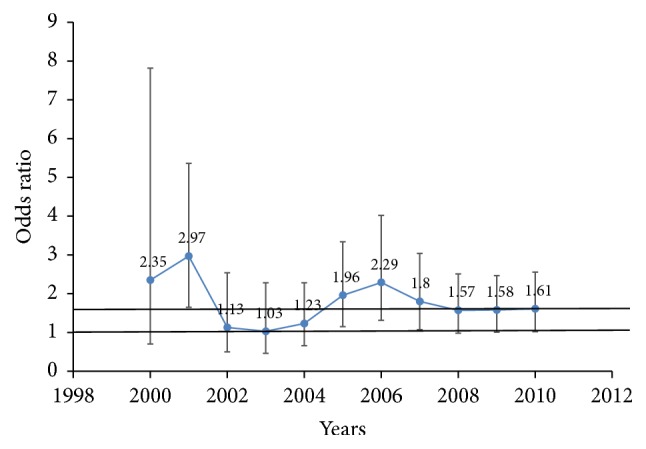
Effects of malaria control in pregnancy during the pre- (2000–2004) and post- (2005–2010) IPTp-SP-policy years on risk of low birth weight deliveries in primigravidae versus multigravidae.

Figure 2 presents the individual year odd ratio changes in both hospitals. The odds of LBW in primigravidae compared to multigravidae were presented graphically across the years with two horizontal lines. The top horizontal line at OR value of 1.7 represents the average OR for the eleven-year period under investigation in both hospitals. The lower horizontal line at an OR of 1 indicates no difference in LBW prevalence between primigravidae and multigravidae. The error bars represent the 95% confidence intervals (CI). Point estimates below the lower line (at OR = 1) indicate a lower prevalence of LBW in primigravidae, and lower error bars beyond this lower horizontal line (at OR = 1) indicate nonsignificant odds of LBW in primigravidae compared to multigravidae. The point estimates were consistently above the lower horizontal line of OR = 1, and more point estimates within the postpolicy years (2005–2010) were above the top horizontal line, which is at an overall average OR of 1.7 across years. All OR point estimates from 2005 to 2010 were significant with lower error bars above the lower line (at OR = 1) (Figure 2).

## 4. Discussion

Higher prevalence and risks of LBW deliveries were recorded in primigravidae, especially during the postpolicy years (2005–2010). This finding probably emphasizes the excess risk of this adverse outcome in primigravidae. Primigravidae have been reported to have increased susceptibility to malaria infections than multigravidae [14] and thus have delivered babies with significantly lower birth weight than multigravidae due to malaria [15]. This birth weight deficit, measured by increased odds of having LBW deliveries due to primiparity, varies in relation to the prevalence of malaria and has been proposed as a simple indicator of malaria transmission and exposure in pregnant women [14]. Thus, the odds ratio (OR) for excess LBW risk in infants of primigravidae has been reported to measure “the excess risk due to malaria alone” while the prevalence of LBW in infants of primigravidae represents “the excess risk due to all causes” [14]. Based on the above premise, it might be inferred that exposure to malaria in pregnant women interestingly increased, albeit nonsignificantly, after the policy change to IPTp-SP in 2005.

Apart from malaria, there are other social and environmental factors that might also affect the prevalence of LBW deliveries. These include, but not limited to, maternal smoking, high blood pressure or preeclampsia, nutritional status of the mother, sex of the baby, number of antenatal clinics visits, maternal anaemia, maternal age, and other maternal infections like HIV [16, 17]. Of all these risk factors, anaemia and preeclampsia were the other LBW risk factors apart from malaria that had the parity-specific effect predominantly affecting primigravidae.

The two variables, maternal anaemia and preeclampsia, were the possible cofounders of the results obtained in this study. Unfortunately, the results of the study could not be adjusted for these possible cofounders as they were not measured due to the retrospective nature of the study and unavailability of these variables in the study instrument used. Maternal anaemia, however, has a closely associated incidence with malaria in endemic areas like Nigeria and is thus unlikely to have any major effect on the sensitivity of the indicator at a population level. Preeclampsia—a pregnancy-specific disorder characterized by hypertension and proteinuria, with or without edema—on the other hand has a very low global prevalence ranging from two to ten percent of live births in developing countries [18]. Records of preeclampsia prevalence within the same geopolitical zones as the facilities used in this study analyses included 1.2% for the period 2009–2011 [19] and 3.3% for the period 2005–2008 [20] for the south-south and southeast geopolitical zones, respectively. It is unlikely that such low prevalence rates would affect the sensitivity of using the risk of LBW in primigravidae as an indicator of malaria exposure during pregnancy.

Another important risk factor contributing to LBW deliveries that might preferentially affect primigravidae is teenage pregnancy. A high proportion of teenage primigravidae in a population would be expected to increase risk estimates. However, an overall low prevalence of women aged less than 18 years of age (1.8%) was recorded in this study and this could not have contributed to the high odds of LBW deliveries observed.

Other risk factors for LBW deliveries like smoking, nutritional deprivation, and sex of the baby do not have such parity-specific effect on fetal growth as malaria and, therefore, might not have possibly contributed to the significantly increased odds of LBW deliveries among primigravidae, especially during the postpolicy period.

A similar study in Malawi evaluating changes in birth weight indicator reported a slight increase in odds of LBW deliveries amongst primigravidae from 2.58 to 2.65 after a policy change from chloroquine to SP. This increase in odds of LBW deliveries in primigravidae, notwithstanding, was accompanied by reductions in the percentage of babies born to primigravidae and multigravidae who had LBW deliveries. The authors, therefore, concluded that since the slight increase in odds of LBW deliveries amongst primigravidae was also accompanied by significant fall in prevalence of LBW deliveries in both gravidae, the change to SP had produced some improvement in birth weight outcomes, even with incomplete regimen [14]. This was in contrast to the findings of this study as the slightly increased odds of LBW deliveries amongst primigravidae were also accompanied by a significant rise in the proportions of LBW deliveries in both gravidae.

As surprising as our results were, poor implementation of effective interventions has been recognized as an important culprit of failure with such interventions. As of 2010, which was the last year of our analyzed data, the national coverage rate of at least two SP doses was very low (13.2%) [21]. Unfortunately, data on the adoption and coverage rates of 2-dose IPTp-SP at the facilities were unavailable during the period of this study.

This study was not without limitations. A major limitation was in terms of unavailability or low quality of data at the health facility. Utilization of individual antenatal care folders of these pregnant women would have yielded data on the other impact indicator, prevalence of maternal anaemia by parity. It also would have been possible to assess the actual use of chloroquine or IPTp-SP among these women and their coverage rates rather than making assumptions of use based on the policy in existence during the years assessed. The study, on the other hand, explored a novel method of analyses using simple routinely collected health facility data to assess the impact of malaria prevention and control in pregnancy.

## 5. Conclusion

The study demonstrated that there was no reduction in the risk of LBW in primigravidae when compared to multigravidae despite the change in policy to IPTp-SP. Rather, the postpolicy period recorded increases in both the excess risk of LBW in primigravidae, compared to multigravidae, and the prevalence of LBW among primigravidae. These are strong pointers that the impact of policy change to IPTp-SP on the prevalence of LBW has not been positive at best.

## Figures and Tables

**Table 1:** Demographic and obstetric characteristics of mother-baby pairs from delivery registers (2000–2010).

Variables	Total
Maternal age group *N*^b^ = 10138 (90.1)	
<18 years	2893 (28.5)
18–34 years	6353 (62.7)
>34 years	892 (8.8)
Gravidity *N*^b^ = 10479 (93.2)	
Primigravidae	3447 (32.9)
Multigravidae	7032 (67.1)
Duration of pregnancy *N*^b^ = 4963 (44.1)	
Term (≥37 weeks <42 weeks)	4751 (95.7)
Preterm (<37 weeks)	125 (2.5)
Postterm (≥42 weeks)	87 (1.8)
Delivery method *N*^b^ = 10850 (96.5)	
Vaginal delivery	8932 (82.3)
Caesarean section (CS)	1918 (17.7)
Baby sex *N*^b^ = 10797 (96.0)	
Female	5235 (48.5)
Male	5559 (51.5)
Baby condition at birth *N*^b^ = 11249 (100)	
Alive	11050 (98.2)
Dead/stillborn	128 (1.1)
Intrauterine fetal death (IUFD)	70 (0.6)

^b^ *N* represents valid cases available, and the figure in a bracket is the percentage of valid cases available for analyses per variable.

**Table 2:** Changes in LBW prevalence and risk (OR) before policy change (2000–2004) and after policy change (2005–2010).

Variables	Before policy change	After policy change	*p*
*Total (BSH & GHC)*			
LBW			
All gravidae	165 (4.7)	409 (6.3)	
Primigravidae	**65 (6.3)**	**193 (8.6)**	**0.031**6^**∗**^
Multigravidae	**100 (4.0)**	**216 (5.1)**	**0.046**3^**∗**^
Odd ratio^a^			
All gravidae	**1.62 (1.17**–**2.23**)^**∗**^	**1.74 (1.43**–**2.13**)^**∗**^	0.7075

^a^Odds of primigravidae having lower birth weight babies than multigravidae; *p: p* value for chi-square and *t*-test using GraphPad software analysis of the impact of the change in policy; ^*∗*^significant at *p* < 0.05.
